# Molecules of Silence: Effects of Meditation on Gene Expression and Epigenetics

**DOI:** 10.3389/fpsyg.2020.01767

**Published:** 2020-08-11

**Authors:** Sabrina Venditti, Loredana Verdone, Anna Reale, Valerio Vetriani, Micaela Caserta, Michele Zampieri

**Affiliations:** ^1^Department of Biology and Biotechnology “Charles Darwin”, Sapienza University, Rome, Italy; ^2^Institute of Molecular Biology and Pathology, National Council of Research (CNR), Rome, Italy; ^3^Department of Experimental Medicine, Sapienza University, Rome, Italy

**Keywords:** meditation, silence, epigenetics, epigenetic marks, gene-expression, mindfulness

## Abstract

Many studies have consistently demonstrated an epigenetic link between environmental stimuli and physiological as well as cognitive responses. Epigenetic mechanisms represent a way to regulate gene activity in real time without modifying the DNA sequence, thus allowing the genome to adapt its functions to changing environmental contexts. Factors such as lifestyle, behavior, and the practice of sitting and moving mindful activities have been shown to be important means of environmental enrichment. Such practices, which include mindfulness meditation, Vipassana, Yoga, Tai Chi, and Quadrato Motor Training, have been reported to positively impact well-being. In fact, they can be considered emotional and attentional regulatory activities, which, by inducing a state of greater inner silence, allow the development of increased self-awareness. Inner silence can therefore be considered a powerful tool to counteract the negative effects of overabundant environmental noise, thanks to its power to relieve stress-related symptoms. Since all these positive outcomes rely on physiological and biochemical activities, the molecular and epigenetic mechanisms influenced by different mindful practices have recently started to be investigated. Here, we review some of the findings that could allow us to uncover the mechanisms by which specific practices influence well-being.

## Introduction

According to the most recent definition given by Cavalli and Heard (2019), epigenetics refers to “the study of molecules and mechanisms that perpetuate alternative gene activity states in the context of the same DNA sequence” (Cavalli and Heard, 2019, p. 489). The activity of genomes is deeply impacted by environmental and lifestyle factors that interface with the genetic information. Epigenetic mechanisms do not change the DNA sequence; instead, they generate different and interchangeable structural states that modify gene activity. These mechanisms include interdependent modifications of DNA and of chromatin, the histone protein structure that compacts DNA in the cell nucleus. These modifications can be grouped into three main categories: DNA methylation, modifications of histones, and small non-coding RNAs. Placement of these epigenetic marks is spatially and temporally controlled and exerts gene-expression regulatory functions. For instance, the addition of methyl groups to cytosines causes the condensation of chromatin, which renders the DNA unavailable to the transcriptional machinery leading to gene silencing. Similarly, acetylation and deacetylation of histones loosen and tighten chromatin, respectively, thus creating “open” or “closed” domains of gene activity along the genome. Finally, microRNAs can control the stability and access of mRNAs to the translation machinery, with an impact on protein production (Cavalli and Heard, 2019).

Mindful practices have long been suggested to promote well-being by producing a state of body relaxation and inner silence, i.e., a state of quiet mind and emotions characterized by the absence of recurring thoughts, images, and emotional fluctuations (Ben-Soussan et al., 2019). Inner silence therefore counteracts the negative effects of the increasing environmental noise reported by the World Health Organization (2011). Consequently, a wide variety of mindful practices stemming from oriental traditions have been introduced in western societies following the impelling demand to increase self-awareness, improve health, and ameliorate the quality of daily life. These practices include a spectrum of meditations, both sitting (i.e., mindfulness meditation, Vipassana, breathing attention) and moving (Yoga, Tai Chi, and Quadrato Motor Training), all of which share the common goal of achieving a state of silence of mind, with positive repercussions on emotional regulation and health. The evidence for their effectiveness is growing and promising mind–body practices are emerging as complementary to more conventional therapeutic interventions. In fact, a considerable amount of literature suggests that mind–body activities can alleviate stress-dependent symptoms of various diseases including psychological disorders (e.g., mood and anxiety disorders), inflammatory diseases, aging, and cancer (Abbott and Lavretsky, 2013; Bower et al., 2015; Chételat et al., 2018). However, although it is likely that related outcomes might be mediated by changes in the levels of some humoral, immune, and neurological factors, the molecular mechanisms underlying the benefits of mind–body interventions remain poorly understood. The analysis of human peripheral tissues (e.g., blood and saliva) has started to show that various types of meditation can reduce levels of the stress hormone cortisol and of reactive oxygen species (ROS), as well as stimulate anti-inflammatory cytokines, endorphins, and neurotrophins (Kasala et al., 2014; Pascoe et al., 2017). In addition, some authors have traced the effect of meditation on such effector molecules back to expression changes of the corresponding genes and, more recently, to specific mechanisms that regulate gene expression (Buric et al., 2017; Kaliman, 2019). The above observations raise the intriguing idea that mindful practices influence the body by means of epigenetics. However, at present, because of the relative novelty of the field, a unifying view of the molecular pathways underlying the benefits conveyed by meditation and a direct correlation between inner silence and specific epigenetic signatures is still lacking. In the current mini-review, we intend to give a summary of the most recent advances in the field of molecular and epigenetic effects of inner silence-inducing activities.

## Epigenetics, Stress, and the Importance of Mindful Activities

During the past 15 years, many studies have correlated alterations of epigenetic marks with conditions of physiological and psychological stress (Szyf, 2012; Provençal et al., 2013; Turecki and Meaney, 2016). Altered profiles of whole-genome DNA methylation were found in brain specimens associated with early-life adverse experiences and in different conditions, such as post-traumatic stress disorder (PTSD; Roth et al., 2011; Roth and Sweatt, 2011) and altered parental care (Weaver et al., 2004; Naumova et al., 2012) in animal models, and mood disorders in humans (McGowan and Kato, 2008). In addition, alterations of histone acetylation profiles and of small non-coding RNAs activity have been found in depression (reviewed in Misztak et al., 2018; Yuan et al., 2018, respectively). Genes affected by differential epigenetic regulation range from modulators of the immune response (i.e., cytokines; Provençal et al., 2013) and glucocorticoids in the hypothalamic–pituitary–adrenal (HPA) axis (Tyrka et al., 2016; Argentieri et al., 2017) to neurotrophins such as brain-derived neurotrophic factor (BDNF) and nerve growth factor (NGF) involved in neuroplasticity, learning, and memory (Roth and Sweatt, 2011).

One key feature of epigenetic information is its potential reversibility. For example, interventions aimed at improving life conditions and behavior (i.e., education, exercise, diet, sleep) were associated with changes in DNA methylation profiles (Naumova et al., 2012; Quach et al., 2017). Early studies of environmental enrichment in rodents (i.e., a combination of multisensory/cognitive stimulation and increased physical activity that enhances social interactions and explorative behavior in mice) showed positive association with improved social behavior and coping with stress, elicited by increased histone acetylation in the hippocampus and neocortex as well as increased production of BDNF (Baroncelli et al., 2010). Moreover, environmental enrichment was associated with decreased DNA methylation at glucocorticoid receptor gene promoter (Gapp et al., 2016) and microRNA-mediated up-regulation of BDNF (McCreary et al., 2016).

In humans, in addition to the beneficial effects of an appropriate lifestyle as reported above, mental and emotional health can be ameliorated by the experience of inner silence, which induces a state of equanimity and leads to improved attention and increased relaxation. To this end, many types of meditation techniques that help reach awareness and reflectivity have been proven to be highly effective. It is therefore of great relevance to investigate whether these practices could reproduce in humans the epigenetic effects elicited by environmental enrichment in rodents and, more importantly, whether it would be possible to identify a specific epigenetic signature for the state of inner silence. In fact, identification of meditation-induced epigenetic marks on the genome may provide critical information on epigenetically modified genes and pathways underlying the association between meditation and mental/emotional health amelioration and may help uncover new targets for therapeutic intervention.

### Molecular and Epigenetic Effects of Movement Meditations

#### Yoga, Tai Chi, and Qigong

Yoga, Tai Chi, and Qigong can improve attention, self-control, and mindfulness by helping to achieve inner silence through movement. Several studies have addressed the physiological effects of these practices at the molecular level.

Yoga was shown to improve the redox state of the body by reducing ROS levels, known to cause inflammation and accelerated aging (Dada et al., 2015; Mohammad et al., 2019). Moreover, Yoga can help cope with stress conditions as shown by reduction of serum cortisol *via* the HPA axis (Tolahunase et al., 2017). However, some authors consider the Cortisol Awakening Response (CAR) a more appropriate measure of stress resilience. In the study by Cahn et al. (2017), CAR appeared significantly increased after training. Importantly BDNF, a central regulator of neuroplasticity, was also found to increase following practice both in healthy (Cahn et al., 2017; Tolahunase et al., 2017) and depressed (Naveen et al., 2013, 2016) subjects. Since BDNF can cross the blood–brain barrier, it is possible to assume that peripheral BDNF levels may reflect those in the brain (Cattaneo et al., 2016). These results suggest that Yoga may counteract neurodegenerative processes triggered by various types of stress by reducing cellular aging and preserving neuroplasticity in the brain. Finally, reduced plasma cortisol levels and increased BDNF were associated with cardiovascular health (Pal et al., 2014). Similarly, the practice of Tai Chi and Qigong was shown to improve immune function by reducing plasma inflammatory cytokines (Campo et al., 2015). Moreover, increased blood levels of endorphins and reduced levels of adrenocorticotropic hormone (ACTH) and cortisol (Ryu et al., 1996; Lee et al., 2004) indicate a positive impact on the HPA axis.

Genome-wide approaches to gene activity, using both plasma and blood samples, have started to be employed to elucidate the effects of mind–body activities (reviewed in Buric et al., 2017) on gene modulation. The first microarray analysis of global mRNAs was carried out in a cross-sectional pilot study of Qigong long-term practitioners. Results showed modulation of several sets of genes having common functions, related to enhanced immunity, lower cellular metabolism, and delayed cell death (Li et al., 2005). Subsequent longitudinal observations revealed that Yoga was able to induce rapid gene expression changes in PBMCs (peripheral blood mononuclear cells, Qu et al., 2013). Similarly, differential expression of genes related to type I interferon response and inflammation was reported following daily Yoga in a population of breast cancer survivors (Bower et al., 2014). In addition, a number of microarray studies revealed transcription profile changes following Tai Chi, involving pathways of inflammation, antiviral response, energy and adrenergic activation, in PBMCs (Irwin et al., 2014, 2015; Kinney et al., 2019).

While differential gene expression and protein levels appear, in most cases, correlated and coherent with the observed biological effects, the mechanisms involved are mostly unknown. However, the involvement of epigenetic regulation seems to be the most likely scenario. Nevertheless, research on epigenetic profiles following mindful movement practices is rare, with only two published studies involving Yoga and Tai Chi practitioners. DNA methylation occurs predominantly at CpG sites located at promoter regions that are known to undergo age-related changes (Fraga et al., 2005; Hannum et al., 2013). The first cross-sectional study, by Ren et al. (2012), analyzed the epigenetic effects of Tai Chi on the methylation of 66 sites using saliva samples from experienced practitioners. They observed that 6 CpG sites on different chromosomes showed significant differences between the trainees and controls. Interestingly, these authors reported that the age-related methylation trend at those CpGs was slower in the Tai Chi group *vs* the controls. Since the age-related decline of DNA methylation reflects the gradual deterioration of important regulatory functions of the genome, the authors speculated that the practice of Tai Chi might protect against the age-related decay of the epigenome. Subsequently, a similar cross-sectional study was conducted by Harkess et al. (2016) who focused on the CpG methylation levels of candidate genes involved in immune function, namely, TNF, IL-6, and CRP, in blood samples of chronically stressed women practicing Yoga compared to a waitlist group. Their main result was that Yoga was associated with hypomethylation of the TNF gene while IL-6 and CRP appeared unaffected. Interestingly, the same result was observed when the waitlist group that later participated in the Yoga intervention was analyzed. However, because of the pilot approach of these studies, further research is required to validate the link between DNA methylation and these practices.

#### Quadrato Motor Training

Here, we treat Quadrato Motor Training (QMT) as a separate case because its unique feature, to the best of our knowledge not present in other movement meditations (i.e., Aikido; Ben-Soussan et al., 2019), is the requirement for second-by-second response inhibition. QMT is a specifically structured walking meditation, developed with the purpose of balancing the three fundamental components of the human being – body, cognition, and emotion (Dotan Ben-Soussan et al., 2013; Paoletti et al., 2017). QMT consists of a set of recorded oral instructions that guide the individual to move within the corners of a 50 × 50 cm square drawn on the floor, by making 12 possible movements. QMT was shown to increase attention, reflectivity and creativity, and these outcomes were correlated with enhanced neuroplasticity as detailed below (Dotan Ben-Soussan et al., 2013; Ben-Soussan et al., 2014). Reflectivity is the capacity to exercise introspection consciously and requires suspension from the habitual thought, inward attention, and receptivity toward the experience (Depraz et al., 2000). Each step taken in the QMT square is followed by a time of waiting for the next recorded instruction, obliging the subject to suspend the tendency for habitual movement (response inhibition). Therefore, the practice requires and reinforces a state of sustained divided attention between the cognitive processing and the motor response, to take the correct direction at each step. During the period of silent waiting between two movements, this unique type of attention brings a state of awareness into the experience of movement itself. Therefore, QMT can be conceived as a form of “mindful movement” (Ben-Soussan et al., 2014, 2019; De Fano et al., 2019; Diamond and Ling, 2019).

Electrophysiological and magnetic resonance studies have suggested that QMT can improve reflectivity and creativity by stimulating neuroplasticity processes leading to increased inter- and intra-hemispheric connectivity (Dotan Ben-Soussan et al., 2013; Ben-Soussan et al., 2014, 2015a; Lasaponara et al., 2017; Piervincenzi et al., 2017). Subsequent studies have suggested a link between the QMT-driven neuroplasticity processes and changes in the salivary levels of proBDNF and proNGF (Ben-Soussan et al., 2015b; Venditti et al., 2015; Caserta et al., 2019). In fact, proNGF was found to decrease following 1 month of practice in both adults and children, in association with an improvement of cognitive and metacognitive functions, such as creativity (Venditti et al., 2015). A parallel pilot study reported increased proBDNF following 3 months of practice, and this variation was correlated with an increase in white matter volume in several brain areas, including the corpus callosum, suggesting improved inter-hemispheric connectivity (Ben-Soussan et al., 2015b). A more recent study comparing levels of proBDNF and proNGF showed significantly correlated increases of both neurotrophins after 3 months of practice (Caserta et al., 2019). It is conceivable that these changes represent the link between the cognitive and psychological outcomes and the ongoing QMT-driven increased neuroplasticity. Further studies are now being carried out to answer the question whether these changes reflect differential expression and/or variations of DNA methylation of the corresponding genes.

### Effects of Sitting Meditations

There are multiple approaches to meditation, including Transcendental Meditation (TM), Zen meditation, Vipassana, Buddhist meditation, Sudarshan Kriya (SK), Kirtan Kriya (KK), Pranayama, and others. Notwithstanding this variety, they share the common goal of achieving mindfulness, a state of moment-to-moment non-judgmental awareness of the actual experience, possibly reached through a state of inner silence.

The abovementioned meditations have been widely studied from the molecular point of view. They influence the levels of several metabolites and biomarkers like hormones and neurotransmitters, as well as immune and neuroendocrine factors affected by stress and relevant to disease development and progression (Daube and Jakobsche, 2015; Robert-McComb et al., 2015; Twal et al., 2016; Househam et al., 2017). For example, TM, SK, and Zen meditation were shown to influence levels of cortisol, serotonin, melatonin, epinephrine and norepinephrine, gamma amino butyric acid (GABA), glutamate, and dehydroepiandrosterone (DHEA; reviewed in Kasala et al., 2014). Nevertheless, although several imaging studies demonstrated that meditation promotes neurogenesis in brain areas involved in cognitive and emotional functions (Tang and Posner, 2014; Lee et al., 2018), studies of neurotrophin levels following meditation are scarce. One report shows increased serum BDNF levels, related to antidepressant activity in subjects practicing SK (Pan et al., 2006). Another study reports on increased salivary NGF following Pranayama (Balasubramanian et al., 2015).

Cross-sectional and longitudinal studies of SK, Pranayama, and KK, using mostly blood samples, in both healthy and clinical populations, found transcriptional changes in common pathways involved in oxidative stress, cell death, aging, cell cycle regulation, and immune response (Sharma et al., 2008; Kumar and Balkrishna, 2009; Black et al., 2013). One interesting case study involving two lifelong expert meditators, able to achieve higher states of consciousness, again revealed differential expression of genes involved in metabolism and cell cycle regulation, immune response, stress response, and cell death (Ravnik-Glavač et al., 2012).

Few epigenetic studies involving experienced meditators were conducted to evaluate possible changes in genome-wide DNA methylation profiling at CpG sites. In the first study, Chaix et al. (2017), using PBMCs, analyzed 353 CpG sites whose methylation level is highly correlated with chronological age across tissues and cell types and represent a measure of epigenetic age (DNAm age; Hovarth, 2013). The deviation between the DNAm age and the chronological age provides information regarding the epigenetic aging rate of an individual (Chen et al., 2016). The study, focused on subjects practicing mindfulness and compassion meditation, revealed that the epigenetic aging rate in meditators is significantly decreased as a function of the practice duration (Chaix et al., 2017). In a follow up of this study, the same authors showed that short meditation interventions can influence the methylome of experienced meditators rapidly, at genes associated with immune metabolism and aging (Chaix et al., 2020). The second methylomic approach, by García-Campayo et al. (2018), compared the methylation profiles obtained from circulating lymphocytes of experienced meditators with more than 10 years of experience with those of meditation-naïve subjects, and identified 64 differentially methylated regions, corresponding to 43 genes involved in glucose homeostasis, lipid metabolism, protein folding, neurotransmission, and modulation of inflammatory pathways.

### Effects of Multiple-Meditation Protocols: Mindfulness-Based Stress Reduction

Following the introduction of meditation in Western countries, many combined protocols of mindful practices have been developed and introduced in several clinical contexts. The first ones were the Relaxation Response (RR, Wallace et al., 1971) and the Mindfulness-Based Stress Reduction (MBSR, Kabat-Zinn, 1982). This latter is an 8-week integrated approach, an amalgam of mindfulness meditation, concentrative meditation, breathing exercises, Yoga, autogenic training, and Buddhist philosophy. In recent years many such protocols, combining different techniques to be applied for defined periods of time to various clinical settings, have been developed under various acronyms, such as Mindful Awareness Practices (MAP; Bower et al., 2014), Mindfulness-Based Movement (MBM; Robert-McComb et al., 2015) and more generally standardized as Mindfulness-Based Interventions (MBI; Black and Slavich, 2016). All protocols have become the subject of psychological, physiological, and molecular investigations (Black and Slavich, 2016). Neurotrophins were studied in only a few cases (i.e., BDNF; Dada et al., 2018; Gagrani et al., 2018). Transcriptomic analyses were performed in several longitudinal and mixed design studies in both healthy and clinical populations combining diverse MBI activities (Dusek et al., 2008; Bhasin et al., 2013; Bower et al., 2014; Kuo et al., 2015; Epel et al., 2016). Results showed that, in both long and short-term practitioners, differential transcription occurs in genes involved in metabolism, inflammatory processes, oxidative stress, and DNA damage response. In most cases, these results were correlated with reduced stress and fatigue, decreased depression symptoms, and improved immune response. A few studies dedicated to the transcriptomic analysis of subjects involved in MBSR protocols obtained similar results (Creswell et al., 2012; Ho et al., 2016).

MBSR was also the subject of two epigenetic studies, one focused on histone acetylation levels following the last day-long session of the MBSR program (Kaliman et al., 2014), and the other focused on the CpG methylation of two specific genes (SLC6A4 and FKBP5) as potential biomarkers for depression (Bishop et al., 2018). Kaliman and collaborators showed lower expression of histone deacetylase genes (HDAC 2, 3, and 9) in PBMCs, as well as alterations of global histone H4 acetylation levels, and proposed that the reduction of HDAC expression may represent a potential therapeutic effect of MBSR in depression. Bishop and colleagues reported hypomethylation of the FKBP5 gene in subjects with PTSD responding to the MBSR intervention compared to non-responders who, in contrast, showed increased methylation. These authors speculated that FKBP5 methylation could be a predictive biomarker of the response to MBSR in PTSD.

## Conclusion and Perspectives

Growing evidence suggests that epigenetic changes are a key mechanism by which a stressful environment acts on the genome, causing stable changes in gene expression and in behavior that can mediate maladaptive responses. On the other end, the voluntary practice of meditation can be considered a form of environmental enrichment, equivalent to positive external stimulation. Hence, it appears fundamental to understand whether meditation can elicit epigenetic events able to prevent disease and promote health. Relevant examples of stress-related targets of epigenetic deregulation are genes involved in glucocorticoid signaling, serotonergic signaling, and neurotrophins. Surprisingly, meditation practices seem to act on the same gene targets, such as FKBP5, SLC6A4, and BDNF, and promote endocrinal, neuronal, and behavioral functions. This suggests that the achievement of a state of inner silence through the practice of meditation can prevent or reverse the detrimental effects of a stressful environment (see Table 1). However, it is unclear whether stress and meditation act antagonistically on shared epigenetic mechanisms and, because of the relative novelty of the field, molecular and epigenetic evidence of the effects of mindful activities is still not sufficient to demonstrate a cause–effect relationship. It is conceivable that, by improving the immune system, metabolism, and stress–response pathways, and by promoting neuroplasticity, meditations of several kinds could affect mechanisms of energy saving, promote homeostasis, and potentiate the reciprocal mind and body’s relaxation abilities, with a positive impact on psychology.

**TABLE 1:** Side-by-side summary of the effects of different meditations.

	Moving meditations				Sitting meditations		Multiple meditation protocols
	Yoga	Tai Chi	Qigong	QMT	TM/SK/KK Pranayama	Mindfulness	MBSR
Epigenetics	↓ DNAmet at CpGs of TNF gene	Slower age-related DNAmet at CpGs.	N/A	N/A	N/A	↓ DNAm age at CpGs ≠ DMR	↓HDAC 2,3, 9 ↑DNA met at FKBP5 gene
	CS, Blood	CS Saliva				L, CS PBMCs Lymphocytes	L, CS PBMCs,
	Harkess et al., 2016	Ren et al., 2012				Chaix et al., 2017, 2020; García-Campayo et al., 2018	Kaliman et al., 2014; Bishop et al., 2018
Differential gene expression (Pathways affected)	Inflammation pathways	Inflammation pathways, antiviral response	↑Immunity, ↓Cell metabolism, delayed cell death,	N/A	Oxidative stress, cell death, aging, cell cycle, immune response	N/A	Inflammation pathways, metabolism, oxidative stress, DNA damage
	L PBMCs	L PBMCs	CS PBMCs		L, CS Blood		L, CS
	Qu et al., 2013; Bower et al., 2014	Irwin et al., 2014, 2015; Kinney et al., 2019	Li et al., 2005		Sharma et al., 2008; Kumar and Balkrishna, 2009; Black et al., 2013		Creswell et al., 2012; Ho et al., 2016
Biomolecules	↓ROS levels ↓Cortisol ↓Inflammation markers	↓Inflammatory cytokines ↓Cortisol	↓ACTH ↓Cortisol ↑Endorphins	N/A	↓Cortisol ↑DHEA ↑Serotonin ↑Melatonin	Markers of inflammation, Markers of stress, Cytokines	Markers of inflammation, Markers of stress, Cytokines
					↓Epinephrine		
					↓Norepinephrine		
	CS Blood	L Saliva	CS, L Plasma		L, CS Various fluids	L, CS Various fluids	L, CS Various fluids
	Reviewed in: Dada et al., 2015; Mohammad et al., 2019	Campo et al., 2015	Ryu et al., 1996; Lee et al., 2004		Reviewed in: [Bibr B29][Bibr B50]	Reviewed in Black and Slavich, 2016	Reviewed in Black and Slavich, 2016
Neurotrophins	↑BDNF	N/A	N/A	↑BDNF (3 mo.) ↓NGF (1 mo.) ↑NGF (3 mo.)	↑BDNF ↑NGF	N/A	↑BDNF
	L Serum Naveen et al., 2013, 2016; Cahn et al., 2017; Tolahunase et al., 2017			L Saliva Ben-Soussan et al., 2015b; Venditti et al., 2015; Caserta et al., 2019	CS, L Saliva, Serum Pan et al., 2006 (SK/KK) Balasubramanian et al., 2015 (Pranayama)		CS Serum Dada et al., 2018; Gagrani et al., 2018

*MBSR, Mindfulness-based stress reduction; QMT, Quadrato Motor Training; TM, Transcendental Meditation; SK, Sudarshan Kriya; KK, Kirtan Kriya; L, Longitudinal; CS, Cross-sectional; DMR, Differentially methylated regions; HDAC, Histone deacetylase; PBMC, Peripheral blood mononuclear cell; ROS, Reactive oxygen species.*

We are still far from identifying specific epigenetic markers associated with the state of inner silence, but the pioneering studies conducted so far suggest that this possibility deserves to be further explored. More epigenetics-focused studies will be necessary to understand the mechanistic details of meditative techniques. An increased, thorough understanding of these techniques and their molecular and epigenetic bases will bring us closer to the possibility of introducing them as non-pharmacological approaches to stress-related diseases and psychological disorders.

## Author Contributions

SV wrote the manuscript. MZ, AR, VV, MC, and LV read and revised the manuscript. All authors contributed to the article and approved the submitted version.

## Conflict of Interest

The authors declare that the research was conducted in the absence of any commercial or financial relationships that could be construed as a potential conflict of interest.
